# Loot

**Published:** 1868-11-15

**Authors:** 


### *LOOT.*

### *Action of Mercury.*

At the recent meeting of the British Medical Association in Oxford, Prof. Hughes Bennett read an abstract of the results which had been arrived at by the Edinburgh Committee. The Committee, after a laborious investigation on the action of mercurials on dogs, arrived at the conclusion, that whether administered in large or small doses, the preparations of mercury exert no cholagogue action upon that animal — in fact, that they always diminish the flow of bile. How far this report can serve to throw light upon the action of mercurials on man, is, however, a matter upon which more than one opinion can be held. In the course of their investigations, the Committee have found that mercurials, when administered in large doses to dogs, purge them; and, when in smaller and frequently repeated doses, induce the same group of phenomena which are observed in men under the same circumstances, viz.: fetor of the breath, salivation, and ulcerations of the gums. Having accurately ascertained these facts, the Committee appear to consider that the fact that mercurials fail to increase the flow of bile in the dog, affords an almost positive proof that these drugs do not exert a cholagogue action in the case of man. The experiments supported also the modern view that the diversion of the bile, through a fistulous opening out of the body, does not materially interfere with the intestinal functions, but leads to exhaustion of the body altogether. Dr. B. W. Richardson accepted the report as a model of scientific work, but urged still that mercury did exert a beneficial effect, and that experience confirmed its value. Was it possible, he asked, that mercury acted on the pancreatic gland as it did on the salivary glands, and that it caused an increased pancreatic secretion? Dr. Bennett, in reply, said it was quite possible the pancreatic function was modified under the action of mercury, for, as one of the tables indicated, the pancreas in five cases was reported as very vascular.—*Medical News.*

### *Drinking-Water in Italy a Cause of Stone.*

The *Lancet* (Aug. 15, 1868) cautions tourists against the drinking-water in Italy. "Florence, and indeed all Tuscany, is very ill-supplied with this necessary of life — the water being supersaturated with inorganic, and even effete organic matter. In Florence itself, the impurities in the water-supply are chiefly alkaline, and these combined with the acid red wines universally drank by the population have caused stone and gravel to be widely prevalent. We have it on the authority of a highly intelligent Florentine, of great medical accomplishments, that 80 per cent. of the population are more or less afflicted with these diseases; and English residents, after but after a few weeks' experience of Florence and its water, have found themselves suffering severely in the kidneys and bladder."—*Med. News and Library.*

